# Coexistence of Acute Demyelinating Polyneuropathy and LRP4-Positive Myasthenia Gravis

**DOI:** 10.1155/crnm/2838547

**Published:** 2025-10-14

**Authors:** Halit Fidancı, Sevgi Turhan, Halil Can Alaydın, Ahmet Yusuf Ertürk, İlker Öztürk

**Affiliations:** ^1^Department of Neurology, Division of Clinical Neurophysiology, University of Health Sciences Adana City Training and Research Hospital, Adana, Türkiye; ^2^Department of Neurology, University of Health Sciences Adana City Training and Research Hospital, Adana, Türkiye

**Keywords:** Guillain–Barré syndrome, myasthenia gravis, the low-density lipoprotein receptor-related protein 4

## Abstract

**Introduction:**

Guillain–Barré syndrome (GBS) and myasthenia gravis (MG) are rare autoimmune disorders that may share overlapping features such as ophthalmoparesis, limb weakness, and bulbar symptoms, complicating the differential diagnosis. Coexistence of GBS and MG or chronic inflammatory demyelinating polyneuropathy and MG, particularly the low-density lipoprotein receptor-related protein 4 (LRP4) antibody-positive subtypes, is extremely rare. We present such a case to highlight diagnostic challenges.

**Case Presentation:**

A 46-year-old man presented with distal weakness, sensory loss, facial diplegia, and dyspnea. Nerve conduction studies revealed demyelinating features, and cerebrospinal fluid analysis showed albuminocytologic dissociation. An acute demyelinating polyneuropathy, most likely GBS, was suspected, and clinical improvement was observed following plasmapheresis. Three weeks later, new symptoms including dysarthria and worsening bulbar weakness emerged. Repetitive nerve stimulation showed a decremental response. LRP4 antibodies were positive, confirming MG. The patient improved with intravenous immunoglobulin, corticosteroids, pyridostigmine, and azathioprine.

**Conclusion:**

This case underscores the rare coexistence of acute demyelinating polyneuropathy and LRP4-positive MG. In acute demyelinating polyneuropathy patients with relapsing or worsening symptoms, coexisting MG should be considered. Comprehensive electrophysiological evaluation and antibody testing, including LRP4, are essential for the accurate diagnosis of both conditions.

## 1. Introduction

Guillain-Barré syndrome (GBS) and myasthenia gravis (MG) are rare autoimmune disorders involving the peripheral nervous system and the neuromuscular junction, with incidence rates of 1-2 and 1-3 per 100,000, respectively [1–3]. GBS may present with limb weakness, reduced deep tendon reflexes, and bulbar symptoms such as facial weakness and dysphagia. MG is characterized by muscle weakness, often presenting as fluctuating ptosis or ophthalmoparesis. Differentiation between GBS and MG relies on clinical presentation, electrodiagnostic studies, and laboratory findings [1–3]. Since ophthalmoparesis, limb weakness, and bulbar symptoms can be seen in both MG and GBS, their coexistence may complicate the differential diagnosis. Furthermore, the occurrence of ptosis, usually regarded as typical for MG, in GBS subgroups such as Miller Fisher syndrome, may blur the clinical distinction between these conditions [1–3].

Although these two autoimmune neuromuscular disorders affect different regions, the coexistence of GBS and MG (GBS-MG) has been rarely reported [4–8]. Similar to the reported association between GBS and MG, a relationship between chronic inflammatory demyelinating polyneuropathy (CIDP) and MG has also been documented [9]. To highlight this unusual association, we present a patient initially diagnosed with acute demyelinating polyneuropathy who was subsequently found to have MG, specifically the low-density lipoprotein receptor-related protein 4 (LRP4) antibody-positive subtype.

## 2. Case Presentation

A 46-year-old male patient described sensory symptoms and weakness in the distal lower extremities starting about 1 week before his presentation. He stated that tingling sensory symptoms and weakness developed in the distal upper extremities in addition to the distal lower extremities 2-3 days after the onset of symptoms. There was no history of recent infection, trauma, vaccination, or diarrhea before the onset of symptoms. His medical history included a 15 year history of diabetes mellitus, coronary artery disease, and chronic alcohol consumption. He denied any prior history of ptosis, diplopia, or dysphagia. In his neurological examination, he had significant hypoesthesia in the distal extremities, mild bilateral facial weakness, and dyspnea. Muscle strength was symmetrically graded as 3 in both distal and proximal muscles of the lower extremities and as 4 in the distal muscles of the upper extremities with minimal proximal weakness, according to the Medical Research Council (MRC) scale. Neurological assessment revealed absent deep tendon reflexes. The patient stated that his symptoms showed no fluctuation during the day, which was consistent with the neurological examination findings. He reported mild dyspnea and was able to reach 20 on the single-breath count test. Bladder and bowel functions were preserved. In sensory nerve conduction studies (NCSs), bilateral median, ulnar and sural compound nerve action potentials (CNAPs) could not be obtained. Motor NCSs of the right median, ulnar, and bilateral posterior tibial nerves showed features of demyelination, including prolonged latencies, slowed velocities, and conduction blocks (Table 1). Positive sharp waves and fibrillation potentials, along with a reduced recruitment pattern, were observed in the right first dorsal interosseous and tibialis anterior muscles. Myopathic motor unit action potentials were not present. The protein level in the cerebrospinal fluid was 1198 mg/L with lumbar puncture and no cells were found. GQ1b and GM1 antibodies were found negative. Serum electrolytes, vitamin b12, folate, and vitamin D levels, as well as liver, renal, and thyroid function tests, were within normal limits. The patient underwent plasmapheresis five times. Muscle strength improved to grade 4 in the distal muscles of the lower extremities with minimal proximal weakness, and grade 5 in the upper extremities upon discharge. There was a significant improvement in the patient's facial weakness, and he reported no further dyspnea.

**Figure 1:**
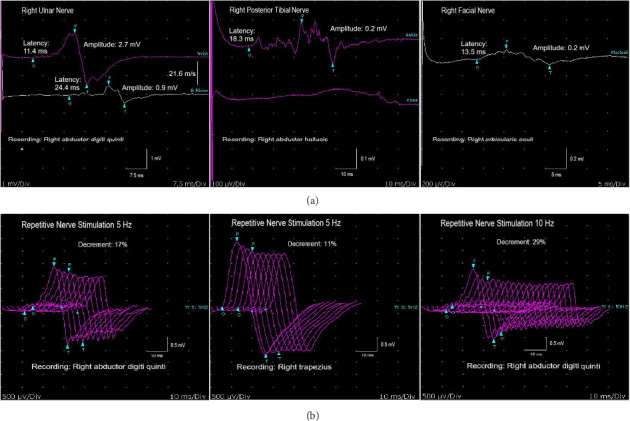
(a) Findings from motor nerve conduction studies. (b) Findings from repetitive nerve stimulation studies.

Approximately 3 weeks later, the patient presented with dysarthria, hypernasal speech, and difficulty swallowing. Compared to the initial visit, the patient exhibited more marked dyspnea and bilateral facial weakness. On reassessment, the patient was able to count only 9-10 on a single-breath. As in the initial study, CNAPs of the median, ulnar, and sural nerves were absent. Motor NCS results are presented in Table 1, and Figure 1(a) illustrates the right ulnar, posterior tibial, and facial motor NCS findings. A decremental response of more than 10% was found in 2, 3, 5, 10 Hz repetitive nerve stimulation (RNS) recorded from the right abductor digiti quinti (ADM) and trapezius muscles (Figure 1(b)). No significant facilitation was observed with exercise in the CMAP recorded from the right abductor pollicis and ADM muscles. Jitter abnormality could not be evaluated, as the patient was unable to tolerate the procedure. Antibodies against the acetylcholine receptor (AchR) and muscle-specific tyrosine kinase were negative, whereas the LRP4 antibody was positive. Thoracic computed tomography revealed no evidence of thymoma. Lumbar puncture was not carried out during the second presentation. The patient was given intravenous immunoglobulin for 5 days. Treatment was initiated with pyridostigmine, oral steroids, and azathioprine. At the examination conducted 3 weeks after discharge, the patient's dysarthria and speech and swallowing difficulties resolved; muscle strength was graded as 4 in the distal lower extremities, while strength in all other muscle groups was normal.

## 3. Discussion

GBS-MG is extremely rare, with an estimated incidence of approximately one in a billion, and has been discussed in only a very limited number of case reports [4–7]. However, this estimate may be low when acute motor axonal neuropathy (AMAN) is considered, as the diagnosis can be missed in some patients. As illustrated in this case, some patients initially considered to have relapses or treatment-related fluctuations may, in fact, have GBS-MG or coexistence of acute-onset CIDP and MG.

The pathophysiological mechanism underlying GBS-MG has not been clearly elucidated; however, it may involve antigens or antibodies, triggered by infection, thymic abnormalities, or other factors, that target both postsynaptic membrane proteins such as AchR and peripheral nervous system components such as sphingomyelin [4, 6, 7]. In light of the current case with LRP4 positivity, one possible explanation is that a trigger or antigen induces antibodies that, via molecular mimicry, cross-react with both peripheral nerves and LRP4. In addition, uncovering the relationship between LRP4 and peripheral nerve regeneration may help explain the association between LRP4-positive MG and GBS [8].

AchR antibodies are commonly identified in patients with MG; in contrast, LRP4 antibody positivity is observed in approximately 1%–3% of all cases and may be associated with a milder clinical course [2, 3]. This may explain the absence of MG-related symptoms in the present case prior to presentation. While GBS-MG has been documented, the absence of AchR antibodies in this case may be attributed to prior plasmapheresis. Although this poses a diagnostic limitation, it is important to note that the antibody testing was performed approximately 3 weeks after the last plasmapheresis session. AchR antibody levels may not have fully returned to baseline within this timeframe, but they are known to begin increasing by the second week postprocedure [10]. Furthermore, although diabetes mellitus and chronic alcohol use could confound the interpretation of NCSs in the diagnosis of GBS, the demyelinating characteristics identified in the NCSs support the presence of an acquired demyelinating polyneuropathy. The findings were suggestive of acute polyneuropathy, as the patient had no prior extremity weakness and reported an acute onset beginning in the lower limbs with subsequent spread. In addition, the prolonged onset latencies and reduced amplitudes observed in the second motor NCS compared to the first support the presence of an acute polyneuropathy. In the setting of chronic polyneuropathy, CMAP amplitudes would be unlikely to decrease to this extent within 4 weeks. While acute-onset CIDP remains a differential consideration, the presence of bilateral facial weakness and dyspnea makes this diagnosis less probable, though not entirely excluded [11]. Therefore, the coexistence of CIDP and MG is also a possibility [9]. At the patient's second admission, lumbar puncture was not performed; however, a decrease in cerebrospinal fluid protein could have suggested GBS.

## 4. Conclusion

We suggest that in patients diagnosed with GBS or acute demyelinating polyneuropathy, when clinical symptoms worsen again following initial improvement after plasmapheresis or IVIG, the possibility of GBS-MG or coexisting acute-onset CIDP and MG should be considered in the differential diagnosis, alongside treatment-related fluctuations or relapses. We also recommend that RNS be performed and that additional antibodies, such as LRP4, are tested along with AchR antibodies, especially in the context of suspected GBS-MG or coexistence of acute-onset CIDP and MG.

## Figures and Tables

**Table 1:** Nerve conduction study findings at initial and second presentations.

NCS	First NCS^∗^			Second NCS^∗^		
	Latency (ms)	Amplitude (μV)	NCV (m/s)	Latency (ms)	Amplitude (μV)	NCV (m/s)
Right F	NA	NA	NA	13.5	200	
Right M						
W	8.13	4400		12.42	4300	
W-E	17.27	1900	28.4	24.73	2400	23.5
Right U						
Wrist	5.94	4200		11.41	2700	
W-BE	16.25	1700	26.2	24.42	900	21.6
Right P						
Ankle	NR	NR	NR	NR	NR	NR
A-BF	NR	NR	NR	NR	NR	NR
Right T						
Ankle	17.58	1000		18.28	200	
A-P	35.23	700	22.6	NR	NR	NR

*Note:* A-BF = ankle-below fibular head segment, A-P = ankle-popliteal fossa segment, F = facial nerve, M = median nerve, P = peroneal nerve, T = posterior tibial nerve, U = ulnar nerve, W = Wrist, W-BE = wrist-below elbow segment, W-E = wrist-elbow segment.

Abbreviations: NA = not available, NCS = nerve conduction study, NCV = nerve conduction velocity, NR = no response.

^∗^The first study reflects the NCS findings at presentation, and the second study those obtained 4 weeks afterward.

## Data Availability

The data that support the findings of this study are available from the corresponding author upon reasonable request.
